# NANOG controls testicular germ cell tumour stemness through regulation of MIR9-2

**DOI:** 10.1186/s13287-024-03724-1

**Published:** 2024-05-01

**Authors:** Ryan P Cardenas, Ahmad Zyoud, Alan McIntyre, Ramiro Alberio, Nigel P Mongan, Cinzia Allegrucci

**Affiliations:** 1 SVMS, Faculty of Medicine and Health Sciences, University of Nottingham, Sutton Bonington Campus, Loughborough, LE12 5RD UK; 2 School of Medicine, Biodiscovery Institute, University of Nottingham, Nottingham, NG7 2RD UK; 3 Centre for Cancer Sciences and Nottingham Breast Cancer Research Centre, Biodiscovery Institute, University of Nottingham, Nottingham, NG7 2RD UK; 4 School of Biosciences, Faculty of Science, University of Nottingham, Sutton Bonington Campus, Loughborough, LE12 5RD UK; 5 Department of Pharmacology, Weill Cornell Medicine, 1300 York Avenue, New York, NY 10065 USA

**Keywords:** Testicular germ cell tumours, NANOG, MIR-9, Differentiation, Pluripotency, Stemness

## Abstract

**Background:**

Testicular germ cell tumours (TGCTs) represent a clinical challenge; they are most prevalent in young individuals and are triggered by molecular mechanisms that are not fully understood. The origin of TGCTs can be traced back to primordial germ cells that fail to mature during embryonic development. These cells express high levels of pluripotency factors, including the transcription factor NANOG which is highly expressed in TGCTs. Gain or amplification of the NANOG locus is common in advanced tumours, suggesting a key role for this master regulator of pluripotency in TGCT stemness and malignancy.

**Methods:**

In this study, we analysed the expression of microRNAs (miRNAs) that are regulated by NANOG in TGCTs via integrated bioinformatic analyses of data from The Cancer Genome Atlas and NANOG chromatin immunoprecipitation in human embryonic stem cells. Through gain-of-function experiments, MIR9-2 was further investigated as a novel tumour suppressor regulated by NANOG. After transfection with MIR9-2 mimics, TGCT cells were analysed for cell proliferation, invasion, sensitivity to cisplatin, and gene expression signatures by RNA sequencing.

**Results:**

For the first time, we identified 86 miRNAs regulated by NANOG in TGCTs. Among these, 37 miRNAs were differentially expressed in NANOG-high tumours, and they clustered TGCTs according to their subtypes. Binding of NANOG within 2 kb upstream of the MIR9-2 locus was associated with a negative regulation. Low expression of MIR9-2 was associated with tumour progression and MIR9-2-5p was found to play a role in the control of tumour stemness. A gain of function of MIR9-2-5p was associated with reduced proliferation, invasion, and sensitivity to cisplatin in both embryonal carcinoma and seminoma tumours. MIR9-2-5p expression in TGCT cells significantly reduced the expression of genes regulating pluripotency and cell division, consistent with its functional effect on reducing cancer stemness.

**Conclusions:**

This study provides new molecular insights into the role of NANOG as a key determinant of pluripotency in TGCTs through the regulation of MIR9-2-5p, a novel epigenetic modulator of cancer stemness. Our data also highlight the potential negative feedback mediated by MIR9-2-5p on NANOG expression, which could be exploited as a therapeutic strategy for the treatment of TGCTs.

**Supplementary Information:**

The online version contains supplementary material available at 10.1186/s13287-024-03724-1.

## Background

Testicular germ cell tumours (TGCTs) are the most prevalent type of solid tumours in young individuals [1]. TGCTs are considered curable malignancies due to their sensitivity to cisplatin-based chemotherapy, however a significant proportion of patients with advanced and refractory disease fail to respond [2]. The quality of life of young patients is also significantly compromised by the long-lasting side effects of chemotherapy [3]; therefore the development of targeted treatments is needed.

TGCTs constitute a heterogeneous group of tumours whose complexity can be traced back to their developmental and cellular origin. Post-pubertal TGCTs derive from primordial germ cells (PGCs) or from gonocytes that are arrested in a stem-like state during maturation. This differentiation block leads to the formation of testicular germ cell neoplasia in situ (GCNIS), which progresses to overt intratubular seminomas (SEM) and invasive SEM. The origin of non-seminoma tumours is less defined, as they appear to derive from the reprogramming of GCNIS or SEM to undifferentiated pluripotent embryonic carcinomas (EC), which can then differentiate into teratomas (TERA), yolk sac tumours (YST) and choriocarcinomas (CH) [4].

However, the molecular mechanisms responsible for the block in differentiation found in TGCTs are still unclear. Gain or amplification of the short arm of chromosome 12 (12p) is one of the most common karyotype abnormalities found in invasive TGCTs [5, 6], and these alterations parallel the selective growth advantage and resistance to differentiation acquired by embryonic stem cells (ESCs) with gain of 12p [7]. These findings suggested that genes at this locus play a critical role in maintaining the undifferentiated state [8]; these genes include the pluripotency gene NANOG, which is expressed in malignant germ cell tumours and essential for maintaining the pluripotency of PGCs [9, 10]. NANOG expression is downregulated when germ cells mature to enter spermatogenesis [11], thus supporting the idea that the expression of master regulators of pluripotency in TGCTs locks PGCs and/or gonocytes in their undifferentiated state. In pluripotent ESCs, NANOG has been shown to regulate microRNAs (miRNAs), which are essential for the regulation of stem cell fate [12]. Indeed, miRNAs regulate stem cells by targeting both pluripotency factors that maintain self-renewal and transcription factors that drive stem cell differentiation [13]. In addition, deficiency in the miRNA biogenesis machinery in ESCs results in failure to extinguish pluripotency factors [14]; similarly, Dicer-knockout mouse germ cells exhibit poor differentiation [15].

In this study, we demonstrate that NANOG regulates a subset of miRNAs that are involved in TGCT pathogenesis. Among these miRNAs, MIR9-2 was identified as a novel tumour suppressor involved in the regulation of TGCT stemness.

## Methods

### Bioinformatics analyses

RNA sequencing data were retrieved from the TGCT Cancer Genome Atlas Project (TGCT-TCGA; dbGaP Study Accession phs000178, open access available from the Genomic Data Commons repository). The datasets were stratified based on patient clinical data (patients *n* = 156) and by gene expression. TGCT subtypes were classified based on dominant histological features (> 80%), with heterogeneous samples (< 80%) classified as mixed. Differential expression was determined using the Robina package of the edgeR statistics package [16]. NANOG normalized log2 median read counts of 12.288 were used as cut-off values for classifying tumours as NANOG high, medium and low, respectively. Differentially expressed genes with a mean log2 (normalized read counts) > 1 and an adjusted P-value < 0.05 (false discovery rate, FDR) were considered significant. Spearman correlation was performed in R to produce a correlation heatmap and a PCA plot. Unsupervised hierarchical clustering was performed using R (v4.2.2) with the hclust package using the Spearman correlation and the complete clustering method. In addition, pre-processed microarray data from the Gene Expression Omnibus (GEO) datasets GSE31824 and GSE18155 [17, 18] were used to analyse the differential expression of TGCT samples compared to that of normal testes. For GSE31824, fold changes were calculated by normalization of TGCT expression to that of the normal testis. For GSE18155, differential expression was measured by the provided ratio of TGCT signal to non-malignant testis tissue. The miRNAs from both the GSE18155 and GSE31824 cohorts were subsequently consolidated to give a unified list (Additional file 1: Table S1). The miRNA names were converted to miRBase version 20 using miRSystem [19] and miRNAs with a minimum 1.5-fold expression change were considered to be differentially expressed. IGV was used to visualize the ChIP-seq location and enrichment in the human genome [20]. ChIP-seq data for the transcription factors NANOG, OCT4 and SOX2 in human ESCs were retrieved from the GEO dataset GSE61475 [21]. For each transcription factor, the ChIP-seq BED files were intersected using Galaxy [22] to give a consolidated BED file for peaks present in all replicates. In addition, BED files from the UCSC browser [23], for sequences 10 kb, 5 kb, 2 kb and 1 kb upstream from all known miRNA sequences were also intersected. The software Venny [24] was used to identify overlapping miRNA candidates.

### RNA sequencing and miRNA target analysis

Total RNA (4 four biological replicates per sample) was extracted using a miRNeasy kit (Qiagen) and RNA sequencing (RNAseq) was performed by Novogene Co., Ltd (Cambridge, UK). RNA integrity and quantitation were assessed using the RNA Nano 6000 Assay Kit of the Bioanalyzer 2100 system (Agilent Technologies, CA, USA). A total of 1 µg of RNA per sample was used as input material for the RNA sample preparations. Sequencing libraries were generated using the NEBNext® UltraTM RNALibrary Prep Kit for Illumina® (NEB) following the manufacturer’s recommendations and index codes were added to attribute sequences to each sample. Clustering of the index-coded samples was performed on a cBot Cluster Generation System using a PE Cluster Kit cBot-HS (Illumina) and library preparations were sequenced on an Illumina 6000 S4 platform and paired-end reads (150 bp) were generated. The raw reads in FASTQ format were first processed using fastp to obtain clean reads by removing reads containing adapter and poly-N sequences and reads of low quality from the raw data. The Q20, Q30, and GC contents of the clean data were calculated and downstream analyses were based on high quality data (QC 93.39–98.06%; error rate = 0.03%). The reference genome (GRCh38/hg38) and gene model annotation files were downloaded from genome website browsers (NCBI/UCSC/Ensembl) directly. Paired-end clean reads were aligned to the reference genome using Spliced Transcripts Alignment to a Reference (STAR v2.5) software (mapping rate 95.9–97.5%). FeatureCounts was used to count the number of reads mapped to each gene and then the RPKM of each gene was then calculated via normalisation to the length of the gene [25]. The RNAseq data are available from the GEO database with accession number GSE232791. Differential expression analysis between two conditions/groups (four biological replicates per condition) was performed with the DESeq2 R package (v2_1.6.3). P values were adjusted using Benjamini and Hochberg’s approach for controlling the FDR. Genes with an FDR < 0.05 were considered to be differentially expressed. Gene Ontology (GO) and pathway analysis was performed with the software Webgestalt [26]. The software package miRWalk v2.0 [27] was used to predict miRNA targets. Several computational algorithms were used to simultaneously construct a list of predicted miRNA targets. The algorithms used were miRDB [28], RNA22-HAS [29], miRANDA [30], and TargetScan [31]. The software Venny [24] was used to create data overlaps.

### Cell culture

The cell lines NTERA-2 clone D1-NT2/D1 (CVCL-3407 [32]), Tera-1 (CVCL_2776 [33]), (a kind gift from Prof. Peter Andrews, University of Sheffield, UK) and HEK-293 cells (CVCL-0045, ECACC) were cultured in DMEM containing 10% foetal calf serum (FCS) and 1% L-glutamine. TCam-2 cells (CVCL-T012 [34], kind gift from Prof. Azim Surani, University of Cambridge) were cultured in RPMI medium supplemented with 10% FCS and 1% L-glutamine. The cell lines were mycoplasma tested using PlasmoTest™ (InvivoGen) and authenticated by genotyping according to the ANSI/ATCC standard ASN-0002 using the Applied Biosystems™ AmpFLSTR™ Identifiler™ Plus PCR Amplification Kit system (Eurofins Genomics). All cell culture materials were obtained from Invitrogen (ThermoFisher) and chemicals were obtained from Sigma Aldrich (Merk), unless otherwise specified.

### Transfections

To create NANOG knockdown cells, TCam-2 cells were transduced with NANOG shRNA or scrambled shRNA Tet-pLKO-puro viral plasmid (Addgene 21,915, Additional file 6: Fig. S1), selected with 0.5 µg/ml puromycin and treated with 1 µg/ml doxycycline. The shRNA primers are listed in Additional file 2: Table S2. For transient NANOG knockdown in NT2/D1 cells, the shRNA plasmid was delivered in 3 rounds of transfections (every 3 days) using the *Trans*IT-X2® delivery system (Mirus). For NANOG expression in HEK293 cells, the cells were transduced with the pSIN-EF2-NANOG viral plasmid (Addgene 16,578, Additional file 6: Fig. S1) and selected with 1.5 µg/ml puromycin. For promoter analysis, the plasmid pGL3 basic plasmid (Promega E1751) expressing the 464 bp genomic sequence fragment upstream of MIR9-2 and the Renilla plasmid (Promega E2231) were co-transfected into cells and luciferase activity was measured after 2 days. The NANOG binding site deletion (MIR9-2 Δ) corresponded to the MIR9-2 locus chr5:+88,666,888 − 88,667,345; Δ (29 bp) chr5:+88,66,7031-88,667,060. For gain-of-function experiments, NT2/D1 and TCam-2 cells were transfected with 25 nM MIR9-2 Mission® miRNA mimic and universal scrambled miRNA (HMI0946 for MIR9-2-5p, HMI0949 for MIR9-2-3p and HMC0002 for universal scrambled; Sigma Aldrich) using the *Trans*IT-X2® delivery system (Mirus). Cells were collected at 3 days post-transfection for RNAseq (4 biological replicates per sample).

### Proliferation, invasion and response to cisplatin treatment

Cell proliferation was assessed by MTT (3-(4,5-dimethylthiazol-2-yl)-2,5-diphenyltetrazolium bromide) assay. Briefly, cells were incubated with 5 µg/ml MTT for 3 h at 37 °C, and the converted formazan dye was solubilized with acidic isopropanol (0.04 M hydrochloric acid in absolute isopropanol). The absorbance was measured using a spectrophotometer at a wavelength of 570 nm with background subtraction at 650 nm.

For cisplatin treatment, cells were seeded at 3,000/well in a 96-well plate to first determine the half-maximal inhibitory concentration IC_50_ (50 µM and 1 µM for TCam-2 and NT2/D1, respectively). After this, sensitization to cisplatin was assessed 3 days after miRNA mimic transfection and after 72 h of treatment.

To analyse cell invasion, cells were first transfected with RNA mimics and then transferred at 3 days post-transfection onto a Falcon Cell Culture Insert with 8.0 μm Pore Transparent PET Membrane fitted into a Companion Plate. A total of 2.5 × 10^5^ cells were seeded onto an insert coated with 300 µg/ml Matrigel (Corning) in serum-free RPMI medium while the same medium supplemented with 10% FCS was placed at the bottom of the plate. Invasion was assessed after 24 h incubation by removing the cells from the top compartment of the insert, fixing the cells with 100% methanol and staining with 0.4% crystal violet in water containing 10% methanol. The cells were imaged using a Nikon Eclipse TE2000-S inverted microscope and images were acquired using Nikon NIS-Elements software.

### Real-time PCR (qRT-PCR)

RNA was extracted using the miRNeasy Mini Kit (Qiagen) and reverse transcribed with the miScript II RT kit (Qiagen). qRT-PCR was performed using the LuminoCt® SYBR® Green qPCR ReadyMix (Sigma Aldrich). Data were analysed using the ΔΔCT method via normalization to the endogenous control genes SNORD75 and ACTB for miRNA and mRNA gene expression, respectively. Endogenous control genes were selected after expression stability was measured across samples using the software BestKeeper [35]. Total RNA for normal testis was obtained from Clontech (catalogue n.636533). The primers used in the study are listed in Additional file 2: Table S2.

### Western blotting

For protein extraction, cells were incubated with RIPA buffer (Cell Signalling Technology) supplemented with protease inhibitor cocktails for 30 min on ice. The cells were then centrifuged at 12,000 x g for 10 min at 4 °C to collect the lysates. After quantification with a Qubit Protein Assay Kit, the proteins were separated using 12% SDS-PAGE (10 µg/lane) and blotted onto a PVDF membrane. The membranes were blocked with 5% skim milk and then incubated overnight at 4 °C with primary antibodies (NANOG: Preprotech 500-P236 0.1 µg/ml; ACTB (beta ACTIN): R&D P37215 0.01 µg/ml). HRP-conjugated secondary antibodies (NA934 and NA931, 1:10,000 dilution, Cytiva) were incubated for 1 h at room temperature and chemiluminescence was detected using the ECL Prime kit (Cytiva) with a LI-COR Odyssey XF imaging system.

### Statistical analyses

The data are presented as the mean ± standard deviation (SD) of biological replicates (at least 3 biological replicates in technical triplicates). One-way and Two-way ANOVA tests were used to analyse samples from more than two groups for comparison. A two-tailed unpaired Student’s t-test was used to analyse samples from two groups for comparison. Statistical analyses were performed using GraphPad Prism 9 software, with *P* < 0.05 indicating statistical significance.

## Results

### NANOG binds miRNAs in TGCTs

**Fig. 1:**
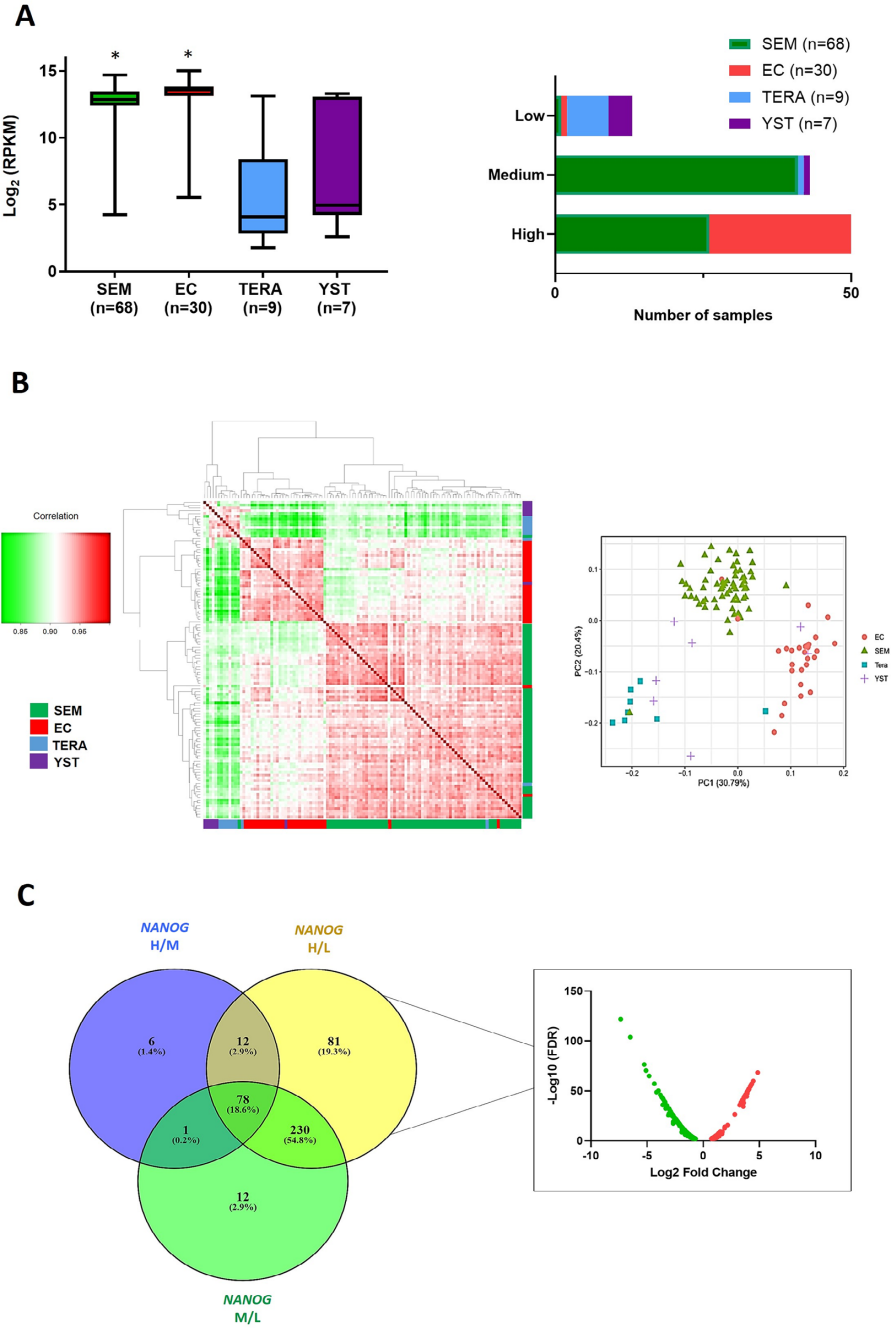
Analysis of NANOG and miRNA expression in TGCTs. (**A**) NANOG expression levels in TCGT subtypes (left) and the number of TCGT subtypes expressing high, medium and low NANOG expression (edgeR, *FDR < 0.05, SEM and EC compared to TERA or YST). (**B**) Spearman correlation heatmap of miRNA gene expression across all samples using unsupervised hierarchical clustering. SEM (green, *n* = 68), EC (red, *n* = 30), TERA (blue, *n* = 9), and YST (purple, *n* = 7) (left). Principal component analysis (PCA) of microRNA gene expression (right). (**C**) Overlap of miRNAs differentially expressed in NANOG H/M/L TGCTs and volcano plot of miRNAs differentially expressed in NANOG H/L (green indicates miRNAs expressed at low levels and red indicates miRNA expressed at high levels in NANOG H/L tumours)

**Fig. 2:**
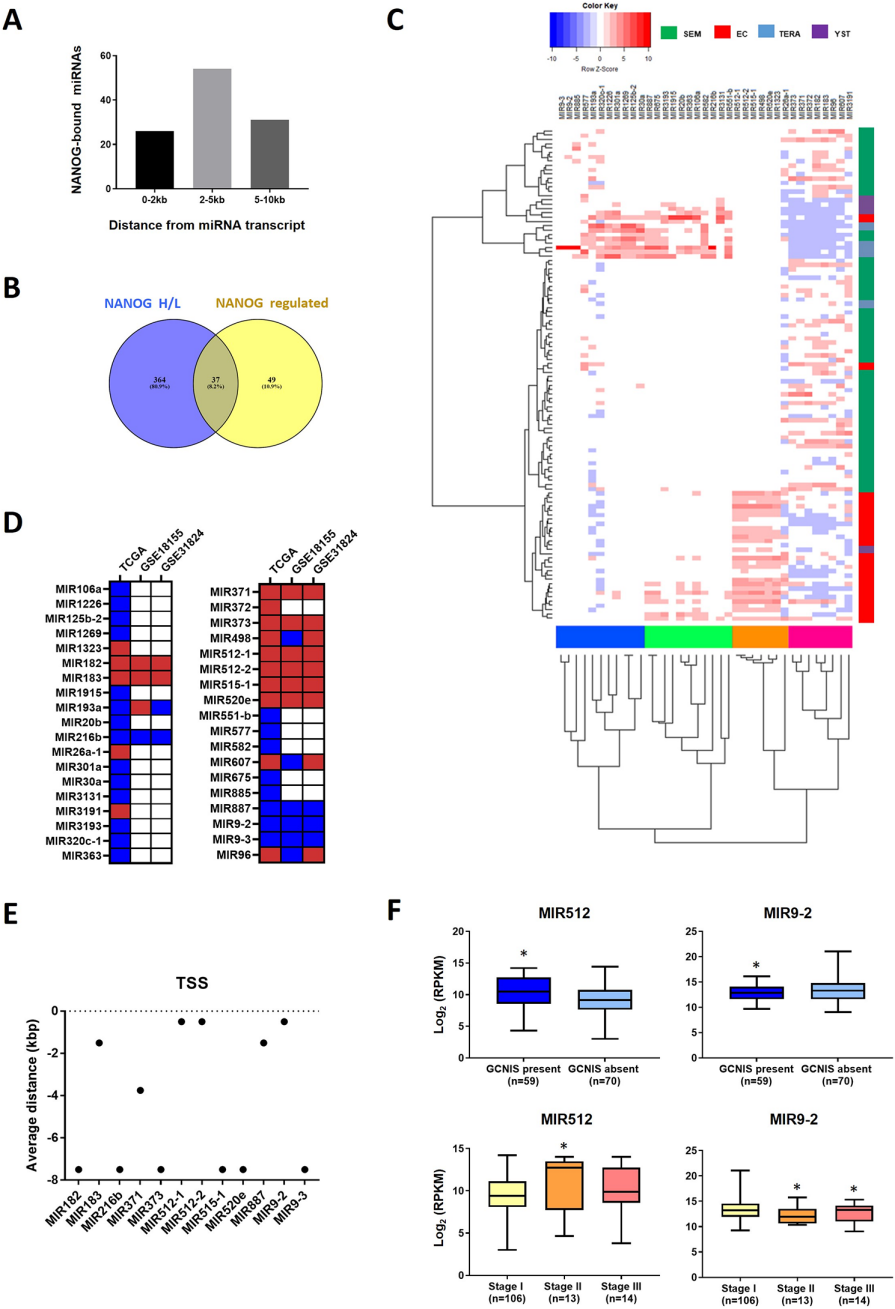
Identification of NANOG-regulated miRNAs and their association with TGCT clinical parameters. (**A**) Number of NANOG-regulated miRNAs with NANOG binding up to 10 kb from miRNA genomic locations. (**B**) Overlap of NANOG-regulated miRNAs and miRNAs differentially expressed in NANOG H/L tumours. (**C**) Unsupervised hierarchical clustering of NANOG-bound/NANOG H/L miRNAs in TGCT subtypes. The blue and green clustering nodes indicate miRNAs expressed in YST and TERA, respectively. The orange and pink clustering nodes indicate miRNAs expressed in EC and SEM, respectively. (**D**) Expression of NANOG-bound/NANOG H/L miRNAs in TGCTs (TCGA) and in microarray studies compared to normal testis (GSE18155, GSE31824). The blue colour indicates miRNAs expressed at low levels and the red colour indicates miRNAs expressed at high levels compared to normal testis. (**E**) Distance of NANOG binding to the genomic location of differentially expressed miRNAs. (**F**) Expression of MIR512 and MIR9-2 in tumours demonstrating the presence or absence of GCNIS (edgeR, *FDR < 0.05, compared to GCNIS absent) and Stage I, Stage II and Stage III disease (edgeR, *FDR < 0.05, compared to Stage I)

To identify miRNAs regulated by NANOG in TGCTs, the TGCT-TCGA dataset was analysed by classifying TGCT subtypes as seminomas (SEM), embryonic carcinomas (EC), teratomas (TERA), or yolk sac tumours (YST). TERA and YST demonstrated the highest variable expression, possibly due to their heterogenous nature. In addition, tumour samples were stratified according to NANOG expression according to tertiles (high (H), medium (M), low (L)). Most of the NANOG-H samples were SEM and EC, whereas TERA and YST mostly represented the NANOG-L samples (Fig. 1A). Unsupervised hierarchical clustering was subsequently performed to determine whether miRNA expression in TGCT tumours could stratify samples into clinical subtypes. Figure 1B shows that SEM and EC clustered together, followed by TERA and YST (left panel). Similarly, principal component analysis (PCA) of miRNA expression grouped the samples into clinical subtypes (Fig. 1B, right panel). The analysis of miRNA expression in tumour samples stratified according to NANOG expression demonstrated that most miRNAs were differentially expressed between NANOG H/L (401 miRNAs), followed by NANOG M/L (321 miRNAs) and by NANOG H/M (97 miRNAs) (Fig. 1C and Additional file 3: Table S3). A great degree of overlap was found between NANOG H/L and M/L (308 miRNAs shared?∼ 96%); therefore, only differentially expressed miRNAs in the NANOG H/L samples were further analysed. There were 115 miRNAs with high expression and 227 miRNAs with low expression in the NANOG-H tumours compared to the NANOG-L tumours (Fig. 1C). We next identified which of these miRNAs were bound by NANOG upstream of their primary transcript genomic location. To this end, we used ChIP-seq data of NANOG binding in pluripotent cells (human ESCs). A total of 86 miRNAs were bound by NANOG up to 10 kb upstream of the miRNA genomic location, with the highest number of binding sites occurring between 2 and 5 kb (Fig. 2A). Many of these miRNAs (48.84%) were also bound by the pluripotency genes OCT4 and SOX2 (Additional file 4: Table S4). Of the 86 miRNAs bound by NANOG, 37 miRNAs were found among the miRNAs differentially expressed in the NANOG H/L tumours; 15 of these miRNAs were highly expressed and 22 were expressed at low levels (Fig. 2B and D). In addition, these shared NANOG-bound/NANOG H/L miRNAs were found to cluster differently in the TCGT samples. Clustering demonstrated high correlation among miRNAs in EC, SEM, TERA and YST, suggesting that the expression of the cluster-specific miRNAs can stratify TGCT tumour samples according to subtype. EC and SEM tumours clustered together, with high expression of MIR512-1, MIR512-2, MIR515-1, MIR498, and MIR520e observed in EC, and MIR371, MIR372, MIR373, MIR182, MIR183, MIR96, MIR607, and MIR3191 in SEM. MicroRNAs expressed in EC and SEM had lower expression in TERA and YST. The remaining miRNAs were highly expressed in TERA and YSK (Fig. 2C).

The expression of NANOG-bound/NANOG H/L miRNAs was also investigated in normal testis tissue using available microarray datasets in the absence of matched normal tissue expression in the TGCT dataset. Of the 37 identified miRNAs, 12 were confirmed to had the same trend of expression (either high or low expression) in TGCTs compared to normal testes. We identified MIR182, MIR183, MIR371, MIR373, MIR512-1, MIR512-2, MIR515-1, and MIR520e exhibiting high expression and MIR216b, MIR887, MIR9-2, and MIR9-3 exhibiting low expression in NANOG H/L tumours (Fig. 2D). These findings indicated possible positive and negative NANOG-driven transcriptional regulation of these miRNAs, respectively. We next analysed the genomic location of the differentially expressed miRNAs and their NANOG binding distance. These included intergenic miRNAs (MIR182, MIR183, MIR373, MIR512-1, MIR512-2, MIR515-1, MIR520e, MIR9-2, and MIR9-3) and intronic miRNAs (MIR371, MIR216b, and MIR887). As miRNA transcription start sites are predicted to be within − 2 kb for intergenic miRNAs and approximately − 4 kb for intronic miRNAs [36], we identified MIR183, MIR512-1, MIR512-2, MIR887, MIR9-2 and MIR371 as candidate miRNAs whose transcription is regulated by NANOG binding (Fig. 2E). However, only the first four identified miRNAs were further analysed given the well-known role of MIR371 in TGCTs [37].

### The expression of NANOG-regulated miRNAs is associated with TGCT clinical parameters

We next evaluated the expression of the selected miRNAs in association with TGCT clinical parameters, including the presence of GCNIS and disease stage. Among the analysed miRNAs, only MIR512 (MIR512-1 and MIR512-2 transcripts) and MIR9-2 were found to be expressed at higher and lower levels, respectively, in tumours with GCNIS. MIR512 expression was also greater in Stage II than in Stage I, whereas MIR9-2 expression was lower in Stages II and III (Fig. 2F). This indicated their clinical relevance due to an association with disease progression. No other candidate miRNAs showed significant association with clinical parameters (Additional file 7: Fig. S2). Although the highlighted differential expression of both MIR512 and MIR9-2 represents a novel finding, we next focused our attention on MIR9-*2* as a potential novel tumour suppressor in TGCTs given the similarity of the seed sequence of MIR512 with that of the pluripotency-associated MIR371-373 cluster [38].

### NANOG functionally regulates MIR9-2 in TGCTs

**Fig. 3:**
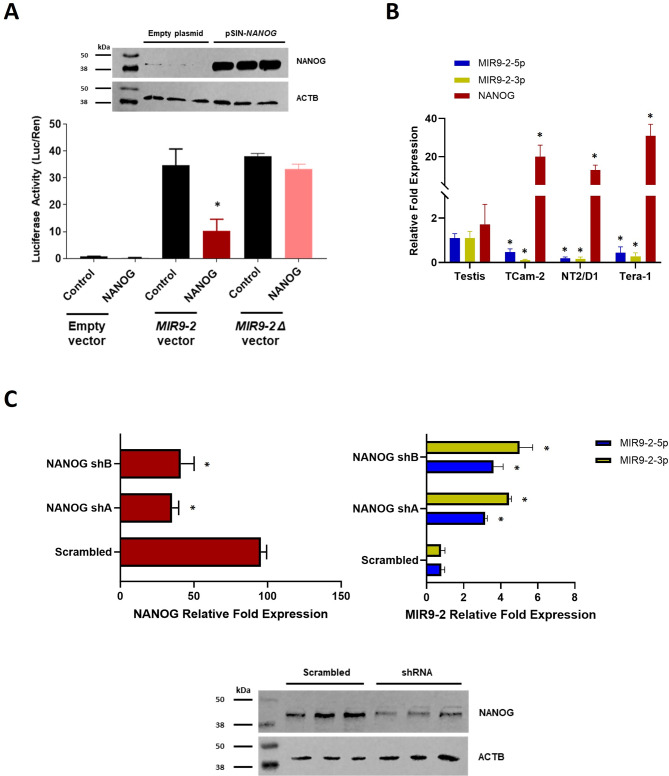
Functional regulation of MIR9-2 by NANOG. (**A**) Cropped western blotting showing exogenous expression of NANOG (pSIN-NANOG) in HEK293 cells compared to that in the empty vector (biological triplicates with expression of ACTB used as a loading control. Full-length blots are presented in Additional file 8: Fig. S3). NANOG expressing cells were subjected to a luciferase reporter assay using MIR9-2 reporter vectors (empty control, MIR9-2, and MIR9-2 Δ deletion, *n* = 3; *P < 0.05 compared to the control; Student’s t-test). (**B**) NANOG expression in TCGT cell lines and normal testes. TCam-2 cells are derived from SEM and NT2/D1, and Tera-1 cells are derived from EC (*n* = 5; *P < 0.05 compared to normal testis; Two-way ANOVA followed by Tukey’s multiple comparisons test). (**C**) qRT-PCR data showing downregulated expression of NANOG after stable knockdown (left) and concomitant upregulated expression of MIR9-2-5p and MIR9-2-3p (right) (*n* = 3); *P < 0.05 compared to the scrambled control; One-way ANOVA and Two-way ANOVA, respectively, followed by Tukey’s multiple comparisons test). Cropped western blotting showing the expression of NANOG in TCam-2 cells transduced with either scrambled or shRNA-NANOG (shA, biological triplicates with expression of ACTB used as a loading control. Full-length blots are presented in Additional file 8: Fig. S3)

To confirm that NANOG binds to the MIR9-2 promoter, we expressed NANOG in HEK293 cells (Additional file 8: Fig. S3) and performed a luciferase assay. The data demonstrated negative regulation of the MIR9-2 promoter, which was lost after deletion of the NANOG binding site sequence, consistent with the bioinformatics analysis (Fig. 3A). We next investigated the function of MIR9-2 in TGCT cell lines and validated its expression using qRT-PCR. To this end, we used TCam-2 cells, derived from SEM tumours [34] and NT2/D1 and Tera-1 cells derived from EC tumours [32, 33]. The expression of the mature sequences MIR9-2-5p and MIR9-2-3p was low in all the cell lines, whereas the expression of NANOG was high, which was consistent with the TGCT RNASeq data (Fig. 3B). Stable knockdown of NANOG in TCam-2 cells (Additional file 8: Fig. S3) resulted in upregulation of both MIR9-2-5p and MIR9-2-3p, thus validating the functional regulation of MIR9-2 by the pluripotency transcription factor (Fig. 3C). However, stable knockdown of NANOG could not be achieved in NT2/D1 cells due to loss of cell viability, which was also affected when prolonged transient knockdown was attempted (Additional file 8: Fig. S3).

### MIR9-2-5p expression reduces TGCT cell proliferation and invasion and sensitises cells to cisplatin

**Fig. 4:**
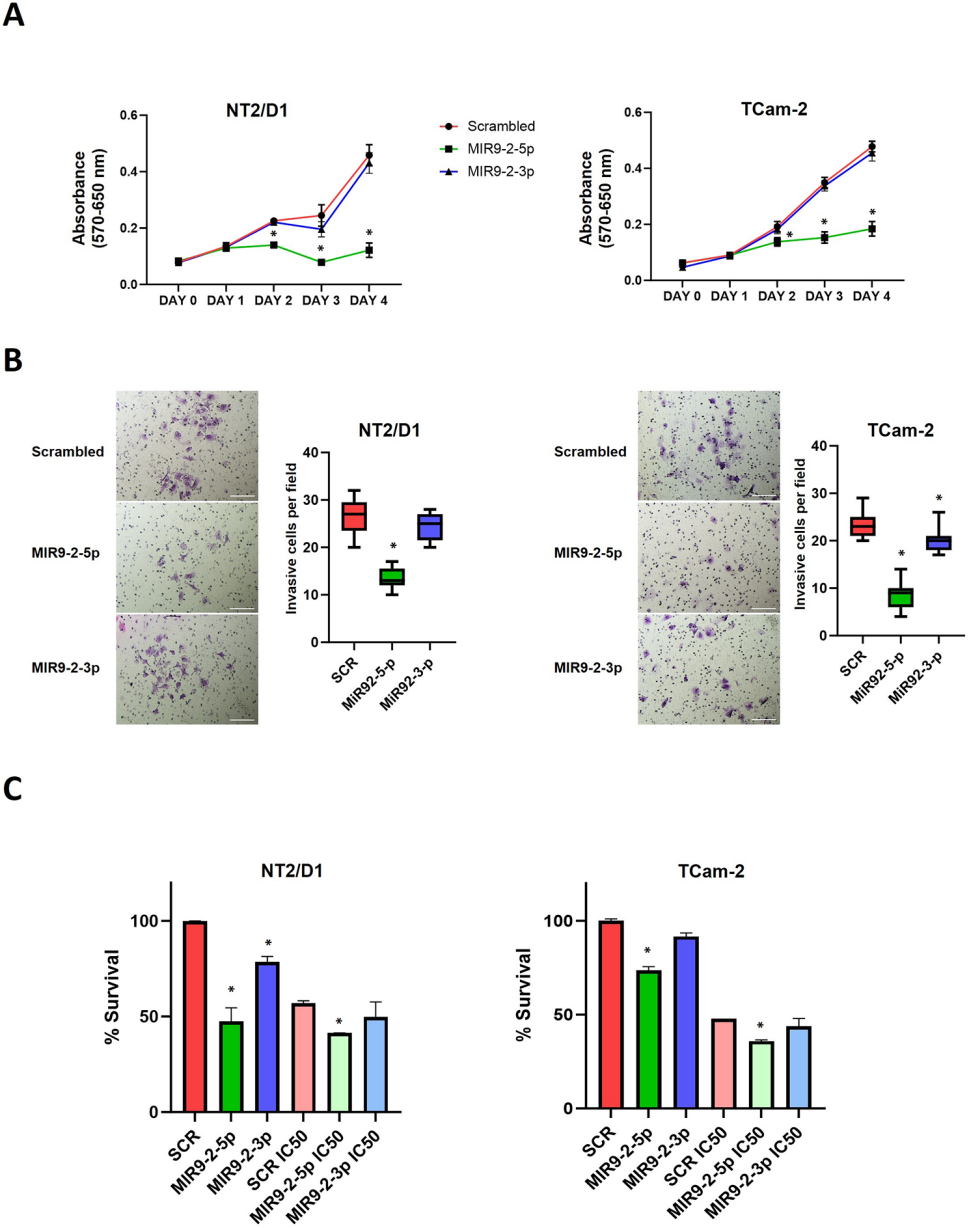
MIR9-2-mediated regulation of TGCT cell proliferation, invasion, and sensitivity to cisplatin. (**A**) Effect of ectopic expression of MIR9-2-5p and MIR9-2-3p induced by transfection of miRNA mimics or miRNA scrambled in NT2/D1 (left) and TCam-2 and cells (right), as measured by: (**A**) cell proliferation assay (*n* = 4; *P < 0.05 compared to scrambled, Two-way ANOVA followed by Tukey’s multiple comparisons test; (**B**) cell invasion assay (*n* = 3, 9–15 fields of view; *P < 0.05 compared to scrambled, One-way ANOVA followed by Tukey’s multiple comparisons test), scale = 500 μm; (**C**) sensitivity to cisplatin (*n* = 3; *P < 0.05 compared to scrambled, Two-way ANOVA followed by Tukey’s multiple comparisons test)

We next assessed the effect of MIR9-2 expression on cell proliferation, invasion, and sensitivity to chemotherapy, which are hallmarks of cancer stemness. The expression of MIR9-2-5p or MIR9-2-3p in NT2/D1 cells and TCam-2 (Additional file 9: Fig. S4) showed that only MIR9-2-5p affected cell proliferation, with a reduction in cell viability measured over 4 days of culture (Fig. 4A). The expression of MIR9-2-5p significantly reduced invasion of both cell lines, with only a modest reduction measured when MIR9-2-3p was expressed in TCam-2 cells (Fig. 4B). In addition, the sensitivity of cells of cisplatin was increased by ∼ 20% and 25% in the NT2/D1 cells and TCam-2, respectively, after expression of MIR9-2-5p (Fig. 4C). Therefore, these functional data demonstrate that the main effect of MIR9-2 is elicited by the transcript MIR9-2-5p, which regulates TGCT stemness features.

### Identification of MIR9-2-5p targets in TGCTs

**Fig. 5:**
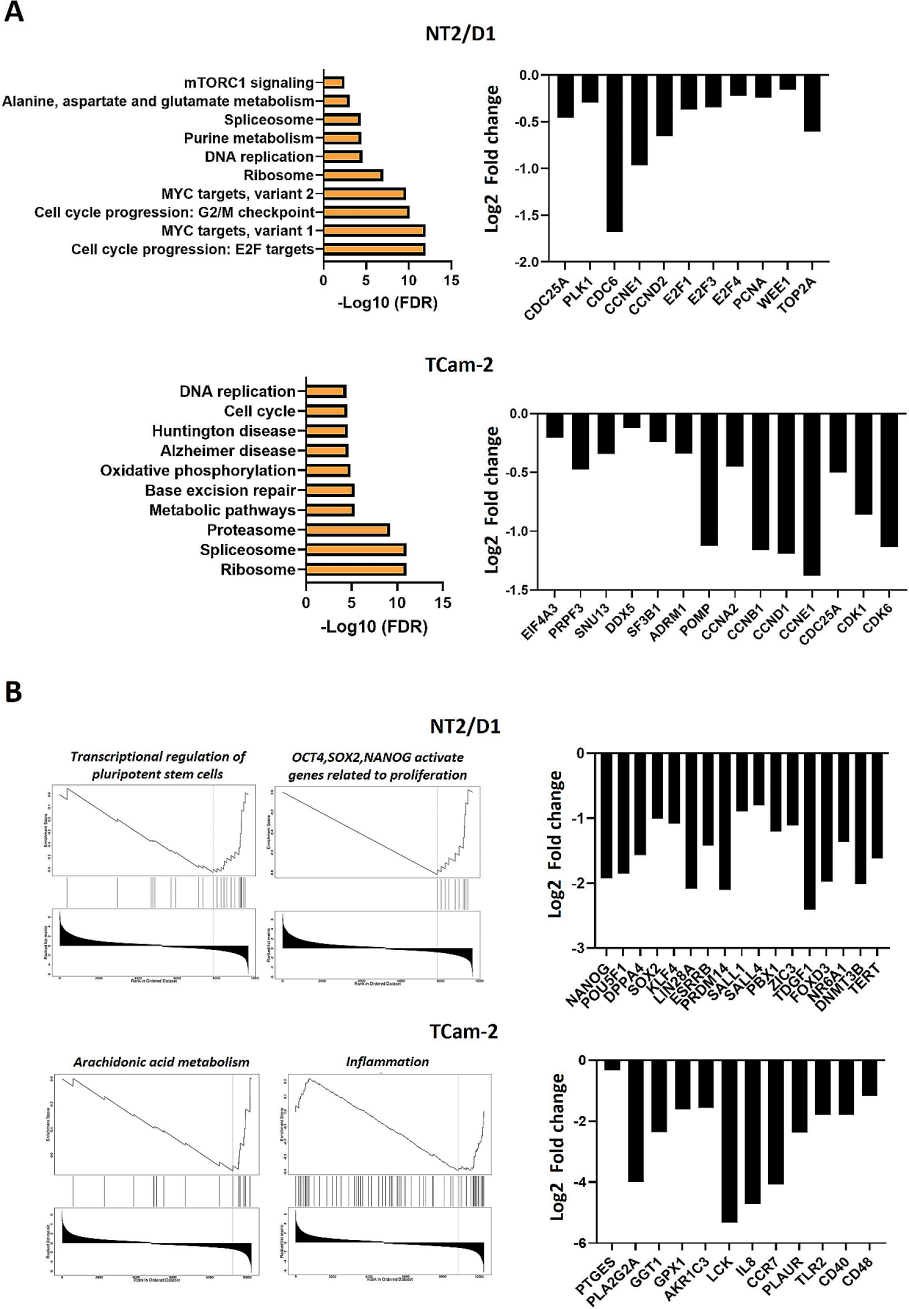
Pathways and gene expression signatures enriched in TGCT cells expressing *MIR9-2-5p* (FDR < 0.05). (**A**) Downregulated pathways (left) and expression of related genes (right, edgeR, *FDR < 0.05 compared to scrambled) (**B**) GSEA enriched signatures (left) and expression of related genes (right, edgeR, *FDR < 0.05 compared to scrambled)

RNAseq analysis of MIR9-2-5p expressing cells revealed a change in the expression signature of both EC and SEM. Pathway analysis revealed the involvement of common processes regulated by MIR9-2-5p in the two TGCT subtypes (Additional file 9: Fig. S4). The pathways associated with genes whose expression was significantly downregulated in EC were related to cell cycle regulation, DNA replication and splicing. These pathways were also highly represented in SEM, confirming the negative effect on cell proliferation observed in functional assays. Additional pathways downregulated in SEM included those related to DNA repair and cell metabolism (Fig. 5A). Gene set enrichment analysis (GSEA) analysis also confirmed the negative enrichment of cell proliferation signatures in both EC and SEM data (Additional file 10: Fig. S5). The downregulation of pluripotency gene signatures was more evident in EC, with the expression of core pluripotency genes, including NANOG, POU5F1/OCT4, DPPA4, SOX2, KLF4, LIN28, ESRRB, DNMT3B, and TERT found to be significantly downregulated. On the other hand, the gene signatures negatively enriched exclusively in SEM were related to arachidonic acid metabolism and inflammatory responses (Fig. 5B).

**Fig. 6:**
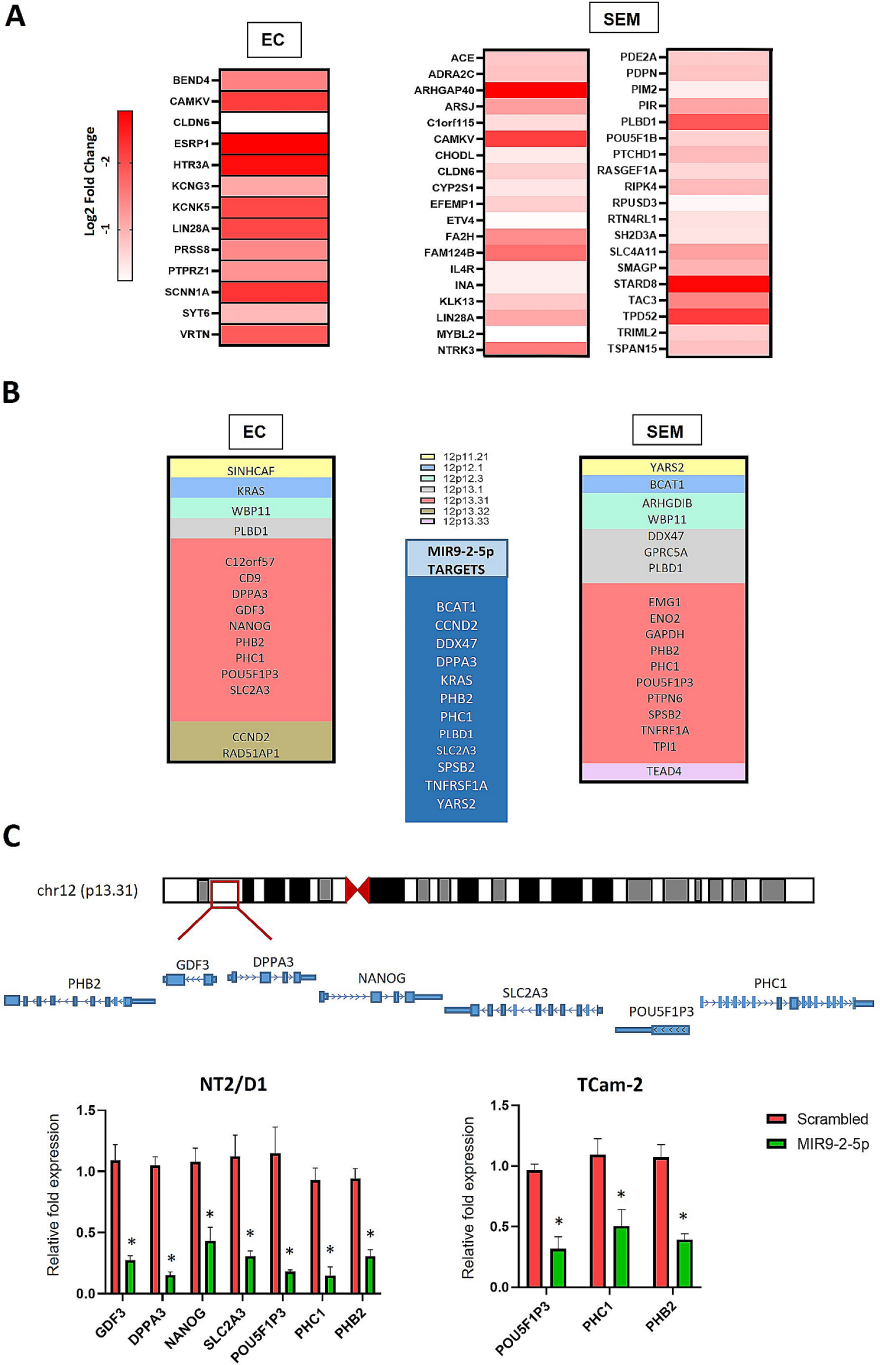
Identification of MIR9-2-5p pluripotency targets. (**A**) Predicted pluripotency targets downregulated in EC or SEM upon MIR9-2-5p expression (edgeR, *FDR < 0.05, compared to scrambled). (**B**) Genes on chromosome 12p expressed in TGCTs and regulated by MIR9-2-5p (direct target genes are highlighted in the blue central box). (**C**) qRT-PCR validation of downregulated genes at the chromosome 12p13.31 locus in EC and SEM cells expressing MIR9-2-5p (*n* = 4; *P < 0.05 compared to the scrambled control, Student’s t-test)

To further validate the functional relationship between NANOG and MIR9-2 we next analysed the expression of predicted MIR9-2-5p targets (Additional file 5: Table S5) by overlapping genes found to be downregulated in cells re-expressing MIR9-2-5p. Pathway analysis identified regulation of cell cycle and splicing in both EC and SEM, further confirming that the gene expression signatures associated with the expression of MIR9-2-5p are due to functional targeting of transcripts (Additional file 11: Fig. S6). As gene expression analyses revealed the downregulation of genes involved in pluripotency, we next tested whether MIR9-2-5p targets included known genes expressed in ESCs and PGCs, as these cell types most closely resemble EC and SEM, respectively. We therefore examined the degree of overlap between genes expressed in pluripotent human ESC [39] and downregulated genes in EC expressing MIR9-2-5p and its predicted targets. This analysis revealed 211 genes in common to EC, many of which were identified by our pathway analysis (data not shown). When the analysis was performed on a subset of 32 pluripotency critical genes, LIN28A was identified as a prominent target. Similarly, analysis of the overlap of genes expressed by SEM, human PGCs and human PGC-like cells [40] revealed 38 genes as direct targets that also included LIN28A and POU5F1B (Fig. 6A and Additional file 12: Fig. S7).

### MIR9-2-5p regulates the expression of genes on chromosome 12p in TGCTs

In line with our hypothesis and our data showing a significant downregulation of pluripotency genes upon expression of MIR9-2-5p, we next examined the expression of genes highly expressed in TGCTs and located on chromosome 12p to investigate a possible coregulatory effect of NANOG and MIR9-2 on the genomic locus. By analysing genes on 12p that were differentially expressed in TCGTs compared to normal testis [11], we found 15 genes downregulated in EC and 18 genes in SEM. Of these, 7 and 8 genes were direct targets of *MIR-9-5p* in the respective tumours (Fig. 6B). Most of the genes were mapped to the 12p13.31 locus, with the pluripotency genes NANOG, DDPA3, GDF3, SLC2A3, POU5F1P3, and PHC1 downregulated in NT2/D1, and POU5F1P3, PHC1 and PHB2 downregulated in TCam-2 cells (Fig. 6C). Importantly, these data confirm the role of MIR9-2-5p in the regulation of pluripotency and suggest the presence of a negative feedback regulatory loop on the chromosome 12p gene cluster containing NANOG. Taken together, these findings point to a critical role for NANOG and MIR9-2-5p in the proliferation and stemness of TGCTs.

## Discussion

Cancer of the testis is generally considered a curable disease, but its clinical management is challenging. The limited availability of predictive/prognostic biomarkers makes treatment of young patients difficult. MiRNAs are considered promising biomarkers for TCGTs because their expression can be specifically detected in tumour tissue and liquid biopsies, with the MIR-371-373 and MIR-302 clusters highly expressed in TGCTs [17, 18] and miR371a-3p currently representing the most promising predictive biomarker [37]. Both the miR302-367 and miR371-373 clusters play critical roles in mouse gonocyte proliferation [41]; therefore, they are considered prominent oncomiRs according to the hypothesis that gonocytes represent the cells of origin of TGCTs [4]. Both clusters are also expressed in human ESCs, and their expression is regulated by pluripotency transcription factors [42]. The expression of pluripotency factors is characteristic of TGCTs, and all invasive TGCTs exhibit a gain of chromosome 12p, with up to 80% of tumours presenting one or more copies of a 12p isochromosome and sequence amplification [6]. One of the key pluripotency genes at this locus is NANOG, which is expressed in PGCs and foetal gonocytes but not in the adult testis [43]. Re-expression of NANOG occurs in GCNIS, SEM and EC, but not in differentiated TGCTs [44], suggesting that reacquisition of pluripotency is a key feature of malignant tumours. In this study, we analysed NANOG-regulated miRNAs to determine their role as prominent epigenetic regulators of pluripotency in testicular cancer. We identified miRNAs whose high expression was associated with different TGCT subtypes suggesting that they may have value in the stratification of patients. Some of these miRNAs were identified in previous TGCT miRNA screens and included MIR182 and MIR183, which were found highly expressed in SEM [18], and MIR520e which was found expressed in non-SEM [45]. Similarly, MIR512 and MIR515 were identified as highly expressed in cisplatin-resistant EC cell lines [46]. Conversely, MIR125b was downregulated in SEM [47]. Our data are consistent with these findings, thus demonstrating the validity of our miRNA discovery pipeline. When the associations of the selected NANOG-regulated miRNAs with clinical parameters were analysed, we found that low expression of MIR9-2 was associated with higher tumour stages and presence of GCNIS, both indicating progression to an invasive tumour phenotype. MIR9-2 is a member of the MIR9 family (MIR9-1, MIR9-2, MIR9-3) that generates the mature MIR9-2-5p and MIR9-2-3p sequences [48]. The role of *MIR9* in cancer is complex, as it can support or suppress tumour development and progression depending on the cancer type and molecular context. For instance, in glioblastoma both MIR9-5p and − 3p act as oncoMIRs and sustain cancer stem cell self-renewal [49]. On the other hand, MIR9 has both tumour suppressive and oncogenic roles in breast cancer as it can repress oestrogen signalling in oestrogen receptor-positive tumours and induce metastasis in ER-negative tumours [50]. In this study, we showed that the expression of MIR9-2 is lower in EC and SEM than in normal testes and that its expression is lower in tumours expressing high levels of NANOG. The expression of MIR9-2 is directly regulated by NANOG binding and we demonstrated that NANOG expression is inversely correlated with MIR9-2 expression in SEM cells. The measurement of this inverse relationship was not possible with EC cells due to loss of cell viability in absence of stable NANOG expression. However, EC cells are considered the malignant counterparts of ESCs [32] and ESCs data have shown that NANOG binds to the proximal promoter of MIR-9 to inhibit MIR-9-mediated differentiation [12]. Therefore, despite the limitation of our study, it is possible that a similar regulation may operate in EC tumours. Although both MIR9-2-5p and MIR9-2-3p were detected at low levels in TGCTs, our data demonstrated that only MIR9-2-5p acts as a tumour suppressor, by suppressing cell proliferation and invasion and increasing sensitivity to cisplatin in both EC and SEM. Increased cell division, migration and resistance to therapy are well defined cancer-stem like features [51]; therefore our data demonstrate that expression of MIR9-2-5p can reduce cancer stemness in TGCTs. This effect was associated with the downregulation of genes involved in cell cycle regulation and DNA replication in both tumour subtypes. Another pathway associated with a reduction in cell proliferation affected in TCam-2 cells expressing MIR9-2-5p was the arachidonic acid metabolism, which is highly active in SEM compared to EC [52]. Arachidonic acid metabolism is linked to the level of inflammation in TGCTs [53], which is associated with advanced pathological stages and immune infiltration. Importantly, inhibition of the pathway by COX and LOX inhibitors as well as by PPAR-ϒ ligands leads to a reduction in TGCT cell proliferation [54], suggesting that MIR9-2-5p has a positive effect on reducing inflammation-related oncogenic pathways in SEM. The downregulation of pluripotency-associated genes was found in both EC and SEM. The expression of LIN28A was reduced in both tumour subtypes and it was found to be a direct target of MIR9-2-5p in mouse ESCs [55]. The expression of the direct target POU5F1B, a transcribed pseudogene of the core pluripotency transcription factor POU5F1 (OCT4), was also downregulated. The other two direct targets SALL4 and ACVR1C were downregulated in EC cells. SALL4 is a transcriptional activator of POU5F1 [56] and therefore could be involved in maintaining the expression of the core pluripotency transcription factors. On the other hand, ACVR1C is a receptor for activin and nodal signalling, the latter being identified as a key pathway involved in the reprogramming and maintenance of pluripotency in EC [57, 58]. Interestingly, other Nodal-related genes including CRIPTO (TDGF1) and LEFTY, were found to be downregulated upon MIR9-2-5p expression in EC. We also found that the expression of MIR9-2-5p induced a reduction in the expression of PRDM14; this protein is needed to stabilize the pluripotency transcriptional network and its downregulation is necessary for the exit from pluripotency both in ESCs and PGCs [59, 60]. During development, MIR-9 is not expressed in PGCs, but its expression increases at later stages (from E12.5) in males, suggesting that MIR-9 is involved in the differentiation of the germline [15]. Interestingly, many of the pluripotency genes downregulated after MIR9-2-5p expression were located on chromosome 12p, which is gained or amplified in TGCTs. In EC, the genes included NANOG, KRAS, CCND2 and SINHCAF, which have been shown to sustain the proliferation of pluripotent cells and play a role in TGCT pathogenesis [61–63]. Other genes, such as DDPA3, GDF3 and SLC2A3 have been linked to ESCs pluripotency and early embryonic development [64–66]. The genes BCAT1 and DDX47 were instead downregulated in SEM and demonstrated to be involved in pluripotency through RAS signalling and in the maintenance of the expression of pluripotency factors, respectively [67, 68]. Common genes in both the EC and SEM cohorts were POUF5F1P3, PHB2 and PHC1. PHB2 and PHC1 are involved in the proliferation and differentiation of stem cells, as well as in the maintenance of pluripotency [69]. Interestingly, PHC1 has been demonstrated to interact with NANOG to stabilize chromatin interactions at the NANOG locus [70]. Importantly, our data showed that most of the genes affected were located at locus 12p13.33, which includes NANOG. These findings suggest that a feedback mechanism operated by NANOG and MIR9-2 maintains the balance between stemness and differentiation in TGCTs.

## Conclusions

Our study demonstrates the key role of NANOG in the regulation of TGCT pluripotency mediated by miRNAs. Our data highlight the role of the novel tumour suppressor MIR9-2-5p, which regulates the proliferation, invasion and stemness of both EC and SEM tumours. Importantly, we have evidence that reduced MIR9-2 expression is associated with tumour progression and that the regulation of NANOG and MIR9-2 could be instrumental for developing therapies for the targeted treatment of TGCTs.

### Electronic supplementary material

Below is the link to the electronic supplementary material.

**Additional file 1: Table S1.** List of miRNAs differentially expressed in TCGTs compared to normal testis obtained from the GSE31824 and GSE18155 datasets

**Additional file 2: Table S2.** Primers used in the study

**Additional file 3: Table S3.** MiRNAs expressed in NANOG high, medium and low tumours

**Additional file 4: Table S4.** NANOG-regulated miRNAs in human ESCs

**Additional file 5: Table S5.** Predicted MIR9-2-5p targets

**Additional file 6: Fig. S1.** Maps of the plasmids used for NANOG expression and knockdown experiments

**Additional file 7: Fig. S2.** Associations of MIR183 and MIR887 expression with TGCT clinical parameters. (**A**) Expression of MIR183 and MIR887 in tumours demonstrating the presence or absence of GCNIS (edgeR, FDR>0.05, compared to GCNIS absent). (**B**) Expression of MIR183 and MIR887 in Stage I, Stage II and Stage III tumours (edgeR, FDR>0.05, compared to Stage I)

**Additional file 8: Fig. S3.** Western blotting of NANOG expression. The dotted lines represent the cropped images shown in Fig. 3. (**A**) Expression of NANOG in HEK293 cells transduced with either pSIN-NANOG or empty plasmid. ACTB expression was used as a loading control to normalize expression (*n* = 3) (*P < 0.05, Student’s t-test). (**B**) Expression of NANOG in TCam-2 cells transduced with either NANOG shRNA (shA) or scrambled plasmids. ACTB expression was used as a loading control to normalize expression (*n* = 3) (*P < 0.05 Student’s, t-test). (**C**) Western blotting of NT2/D1 cells transiently transfected with the Nanog shRNA plasmid. Three rounds of transfections (1st, 2nd, 3rd) were performed (every 3 days of culture) (*n* = 2; UT = Untransfected cells). Images of transfected cells (top) and MTT staining show (bottom) show loss of viability over time

**Additional file 9: Fig. S4.** Analysis of differentially expressed genes in EC and SEM expressing MIR9-2-5p. (**A**) Expression levels of MIR9-2-5p and MIR9-2-3p after mimic treatment (*n* = 3; *P < 0.05, One-way ANOVA followed by Tukey's multiple comparisons test compared to the scrambled control). (**B**) Volcano plots of differentially expressed genes in EC and SEM (edgeR, FDR < 0.05). (**C**) Pathways enriched in both EC and SEM (FDR < 0.05)

**Additional file 10: Fig. S5.** Analysis of differentially expressed genes in EC and SEM expressing MIR9-2-5p (FDR < 0.05). (**A**) GSEA enriched pathways in EC. Negative enrichment is indicated by orange bars whereas positive enrichment is indicated by blue bars. (**B**) GSEA enriched pathways in SEM. Negative enrichment is indicated by orange bars whereas positive enrichment is indicated by blue bars

**Additional file 11: Fig. S6.** Pathway analysis of genes downregulated in EC and SEM that are MIR9-2-5p targets (FDR < 0.05). (**A**) EC pathways. (**B**) SEM pathways. (**C**) Pathways common to both EC and SEM.

**Additional file 12: Fig. S7.** Identification of MIR9-2-5p pluripotency targets. (**A**) Overlap of predicted targets with ESCs genes and downregulated genes in EC. Validation of pluripotency targets LIN28A and POU5F1P3 (*P < 0.05, Student’s t-test). (**B**) Overlap of predicted targets with PGCs genes and downregulated genes in SEM. Validation of pluripotency targets LIN28A and POU5F1P3 (*P < 0.05, Student’s t-test)

## Data Availability

RNA sequencing data are available from GEO (accession number GSE232791; https://0-www-ncbi-nlm-nih-gov.brum.beds.ac.uk/geo/query/acc.cgi?acc=GSE232791). Supporting data are contained in the manuscript as Supporting information. Materials are available from the corresponding author upon reasonable request.

## Abbreviations

CH: Choriocarcinoma
EC: Embryonic carcinoma
ESC: Embryonic stem cell
FDR: False discovery rate
GCNIS: Germ cell neoplasia in situ
GEO: Gene Expression Omnibus
miRNA: microRNA
PGC: Primordial germ cell
SEM: Seminoma
TCGA: The Cancer Genome Atlas
TE: Teratocarcinoma
TGCT: Testicular germ cell tumour
YST: Yolk sac tumour
