# Supplementary figures and images for: NANOG controls testicular germ cell tumour stemness through regulation of MIR9-2
Source: Stem Cell Res Ther. 2024 May 1;15:128. doi: 10.1186/s13287-024-03724-1 (PMC11062916; doi:10.1186/s13287-024-03724-1)

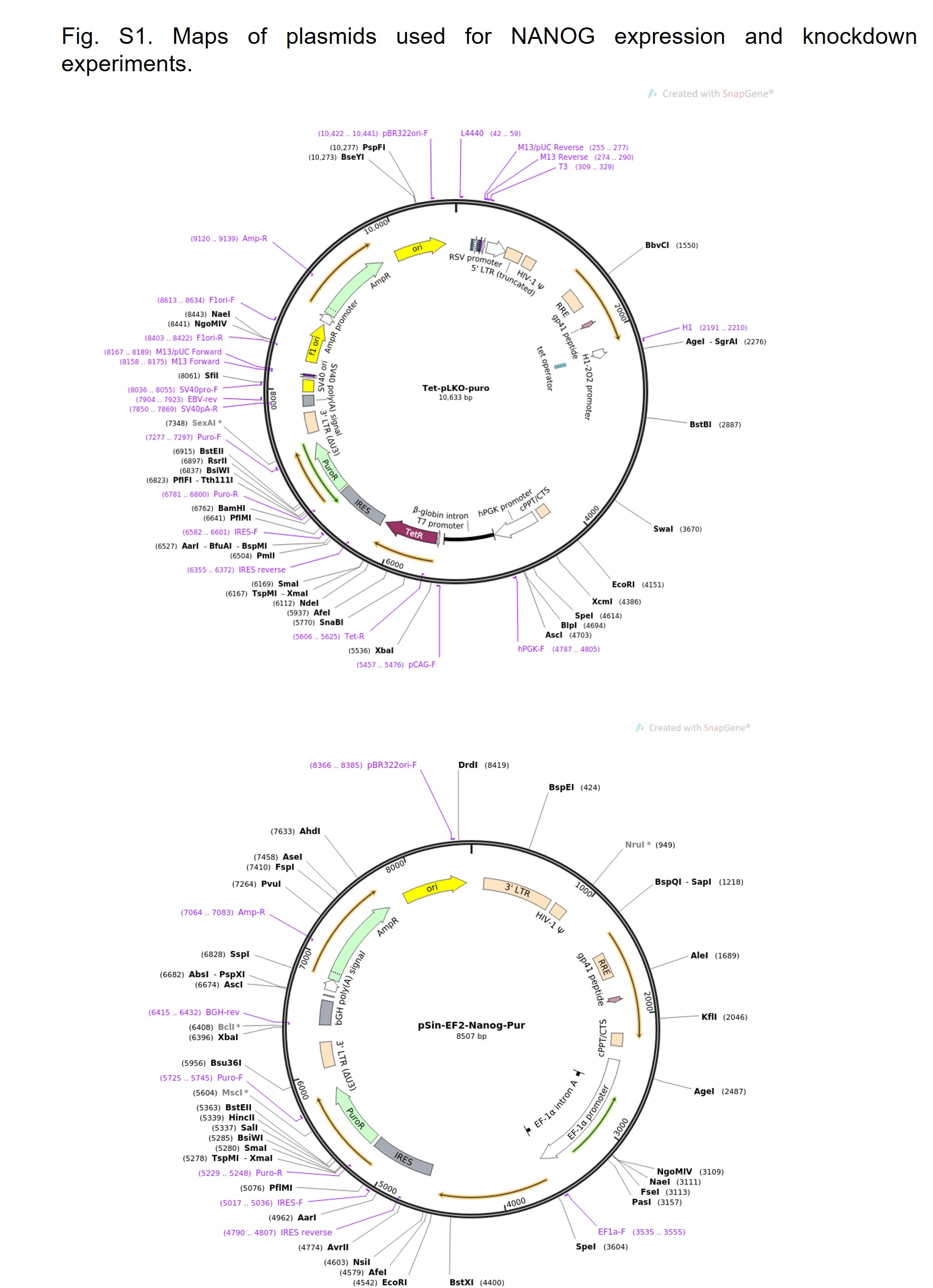

Supplement: Supplementary file 6 — Additional file 6: Fig. S1. Maps of the plasmids used for NANOG expression and knockdown experiments [file 13287_2024_3724_MOESM6_ESM.png]

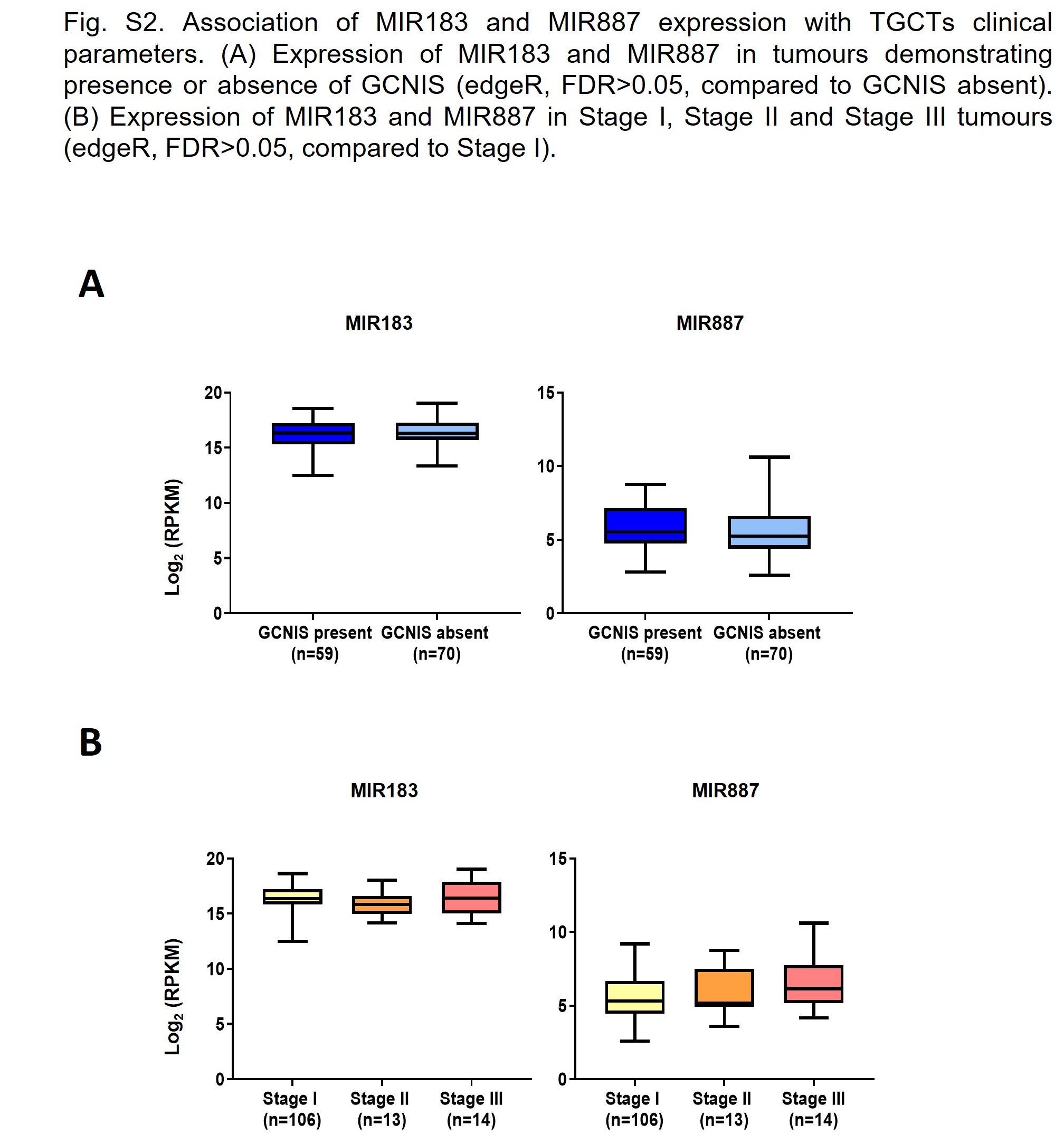

Supplement: Supplementary file 7 — Additional file 7: Fig. S2. Associations of MIR183 and MIR887 expression with TGCT clinical parameters. (A) Expression of MIR183 and MIR887 in tumours demonstrating the presence or absence of GCNIS (edgeR, FDR>0.05, compared to GCNIS absent). (B) Expression of MIR183 and MIR887 in Stage I, Stage II and Stage III tumours (edgeR, FDR>0.05, compared to Stage I) [file 13287_2024_3724_MOESM7_ESM.png]

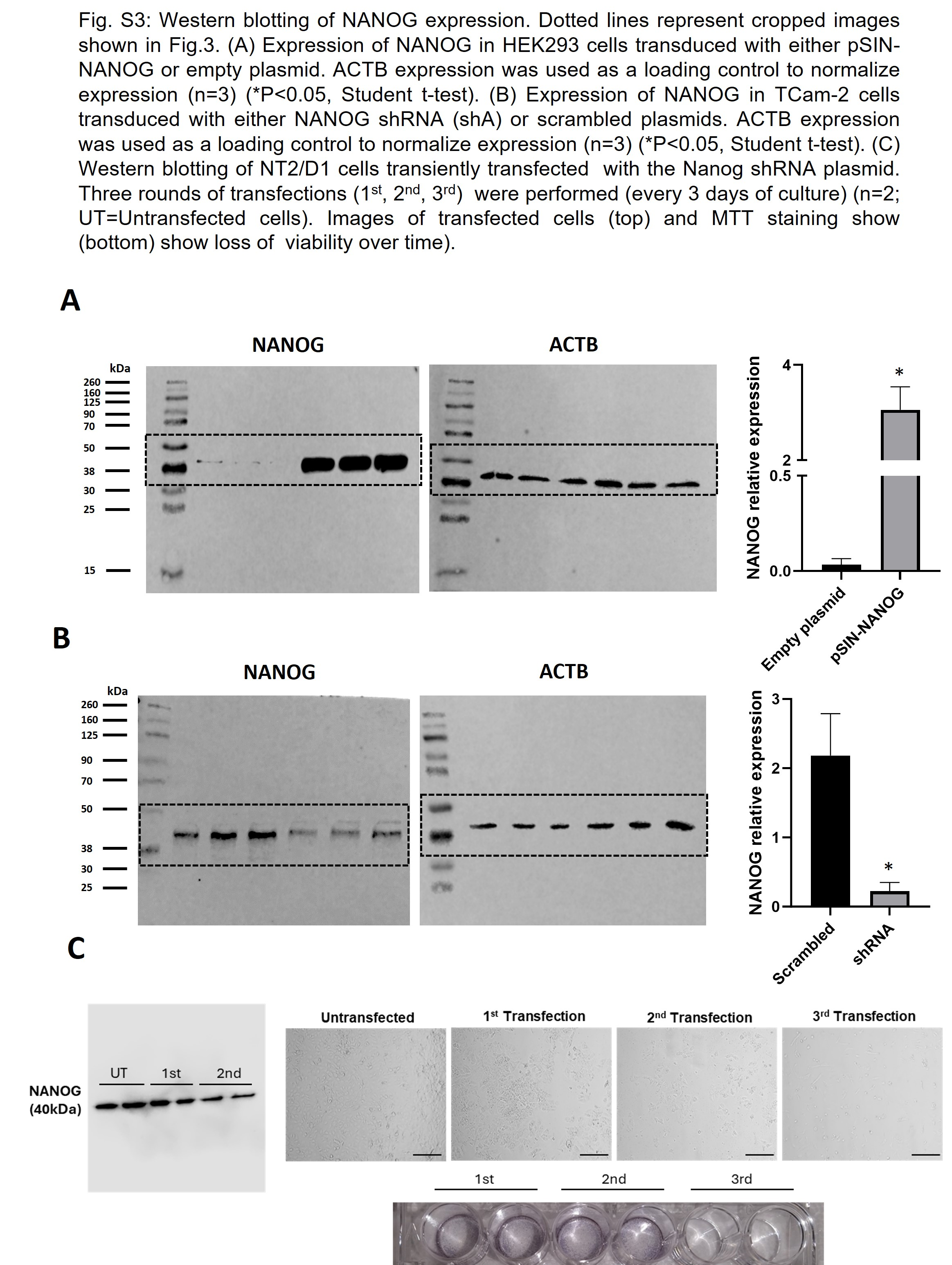

Supplement: Supplementary file 8 — Additional file 8: Fig. S3. Western blotting of NANOG expression. The dotted lines represent the cropped images shown in Fig. 3. (A) Expression of NANOG in HEK293 cells transduced with either pSIN-NANOG or empty plasmid. ACTB expression was used as a loading control to normalize expression (n = 3) (*P < 0.05, Student’s t-test). (B) Expression of NANOG in TCam-2 cells transduced with either NANOG shRNA (shA) or scrambled plasmids. ACTB expression was used as a loading control to normalize expression (n = 3) (*P < 0.05 Student’s, t-test). (C) Western blotting of NT2/D1 cells transiently transfected with the Nanog shRNA plasmid. Three rounds of transfections (1st, 2nd, 3rd) were performed (every 3 days of culture) (n = 2; UT = Untransfected cells). Images of transfected cells (top) and MTT staining show (bottom) show loss of viability over time [file 13287_2024_3724_MOESM8_ESM.png]

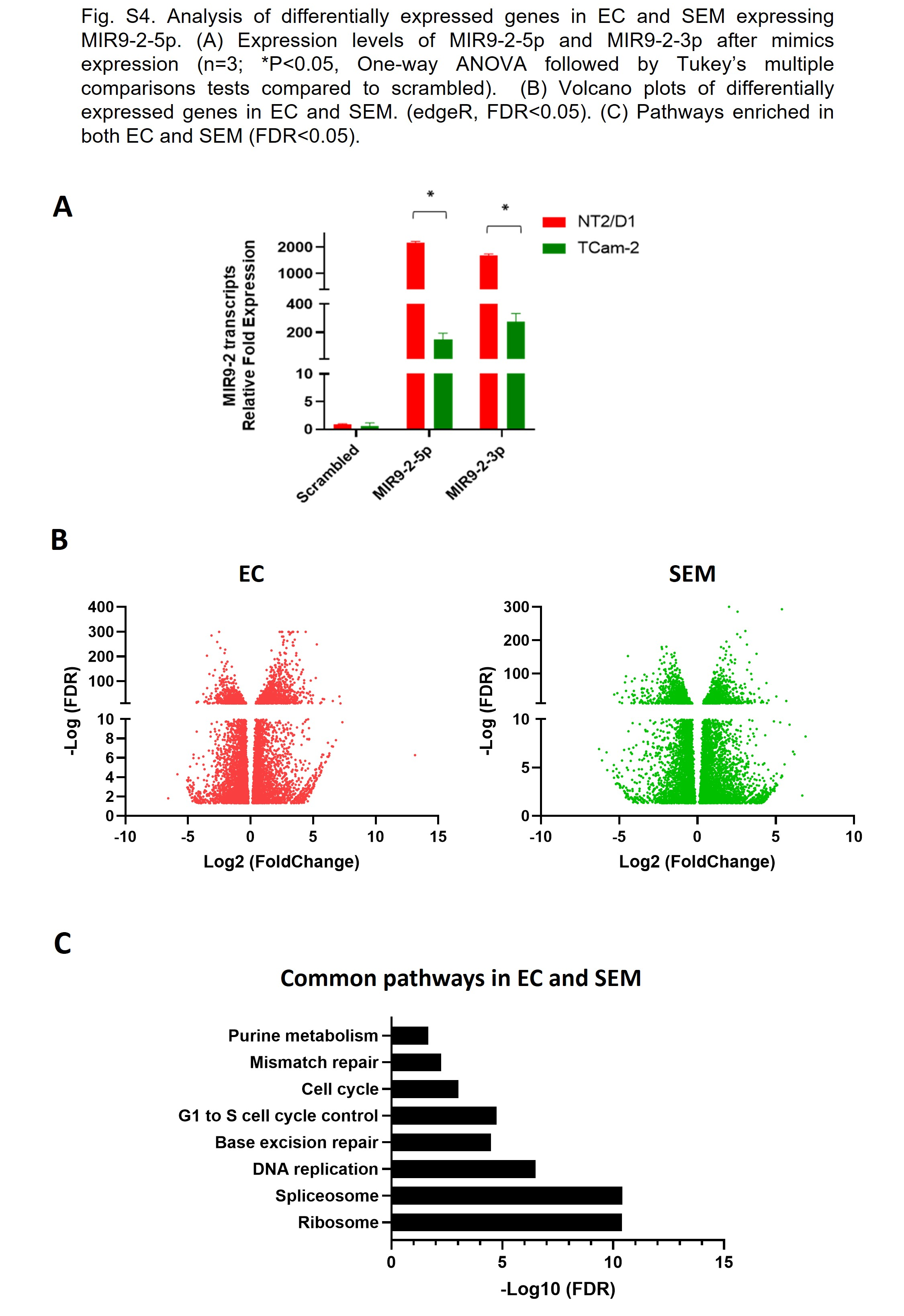

Supplement: Supplementary file 9 — Additional file 9: Fig. S4. Analysis of differentially expressed genes in EC and SEM expressing MIR9-2-5p. (A) Expression levels of MIR9-2-5p and MIR9-2-3p after mimic treatment (n = 3; *P < 0.05, One-way ANOVA followed by Tukey's multiple comparisons test compared to the scrambled control). (B) Volcano plots of differentially expressed genes in EC and SEM (edgeR, FDR < 0.05). (C) Pathways enriched in both EC and SEM (FDR < 0.05) [file 13287_2024_3724_MOESM9_ESM.png]

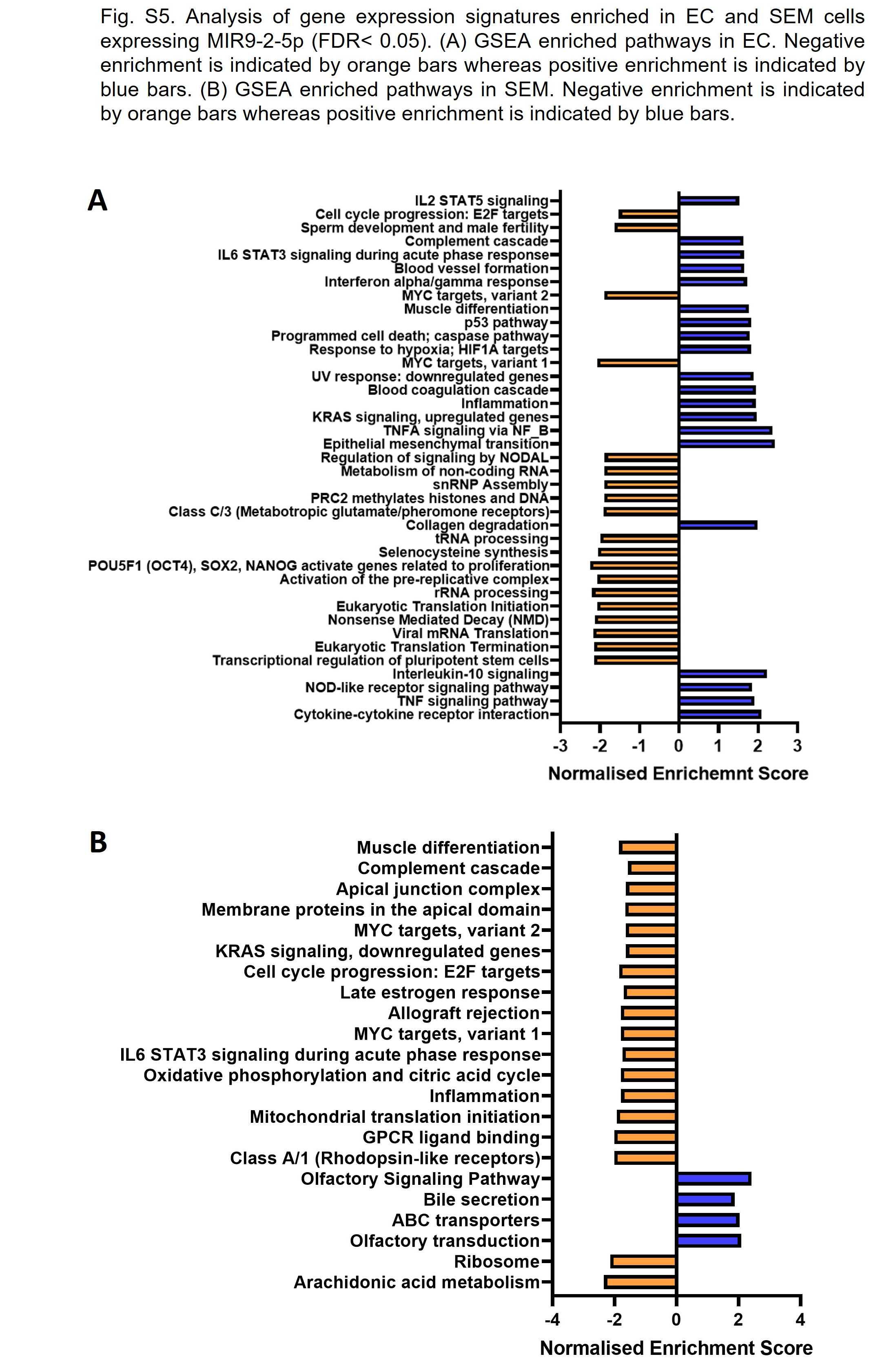

Supplement: Supplementary file 10 — Additional file 10: Fig. S5. Analysis of differentially expressed genes in EC and SEM expressing MIR9-2-5p (FDR < 0.05). (A) GSEA enriched pathways in EC. Negative enrichment is indicated by orange bars whereas positive enrichment is indicated by blue bars. (B) GSEA enriched pathways in SEM. Negative enrichment is indicated by orange bars whereas positive enrichment is indicated by blue bars [file 13287_2024_3724_MOESM10_ESM.png]

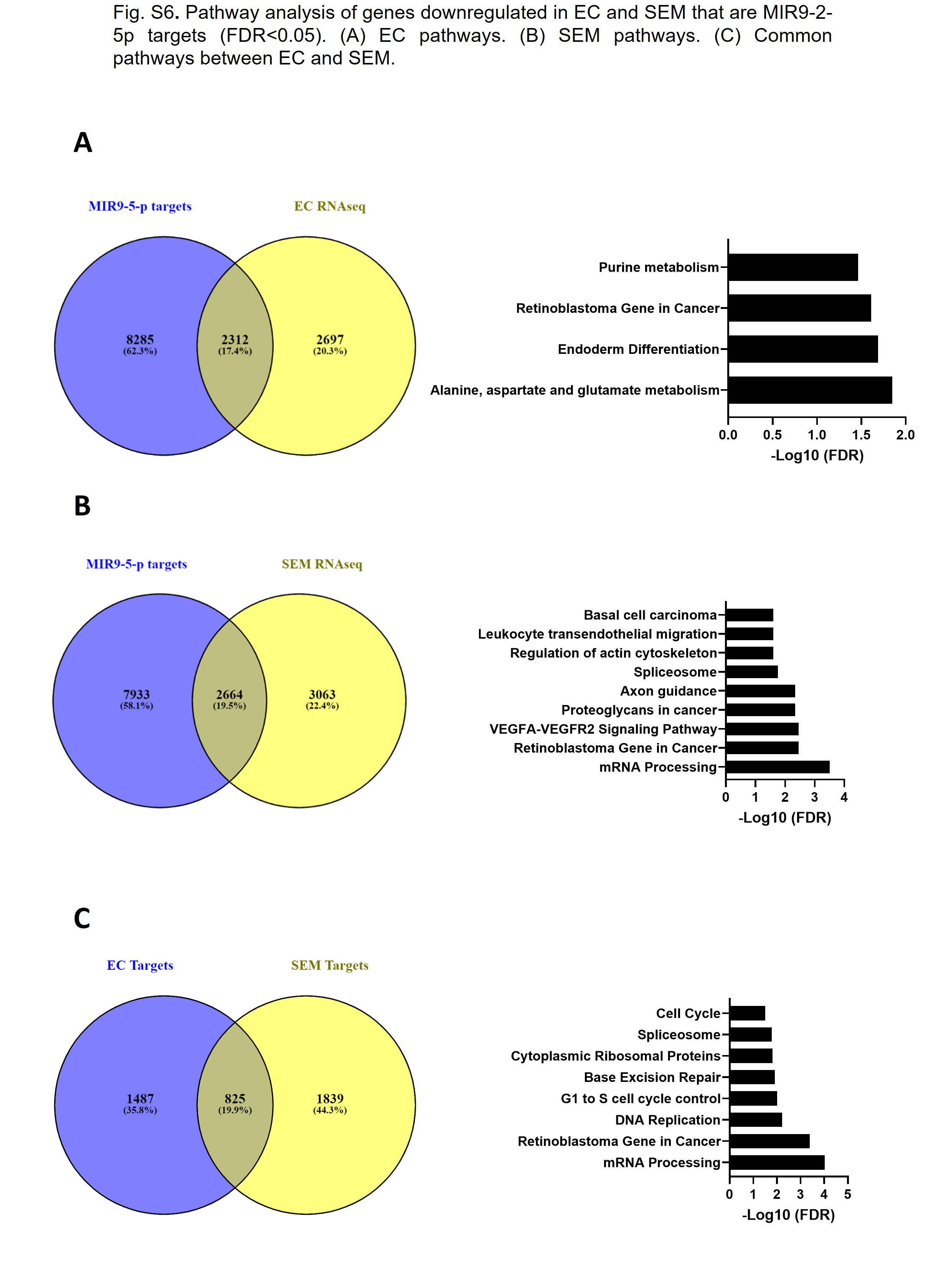

Supplement: Supplementary file 11 — Additional file 11: Fig. S6. Pathway analysis of genes downregulated in EC and SEM that are MIR9-2-5p targets (FDR < 0.05). (A) EC pathways. (B) SEM pathways. (C) Pathways common to both EC and SEM. [file 13287_2024_3724_MOESM11_ESM.png]

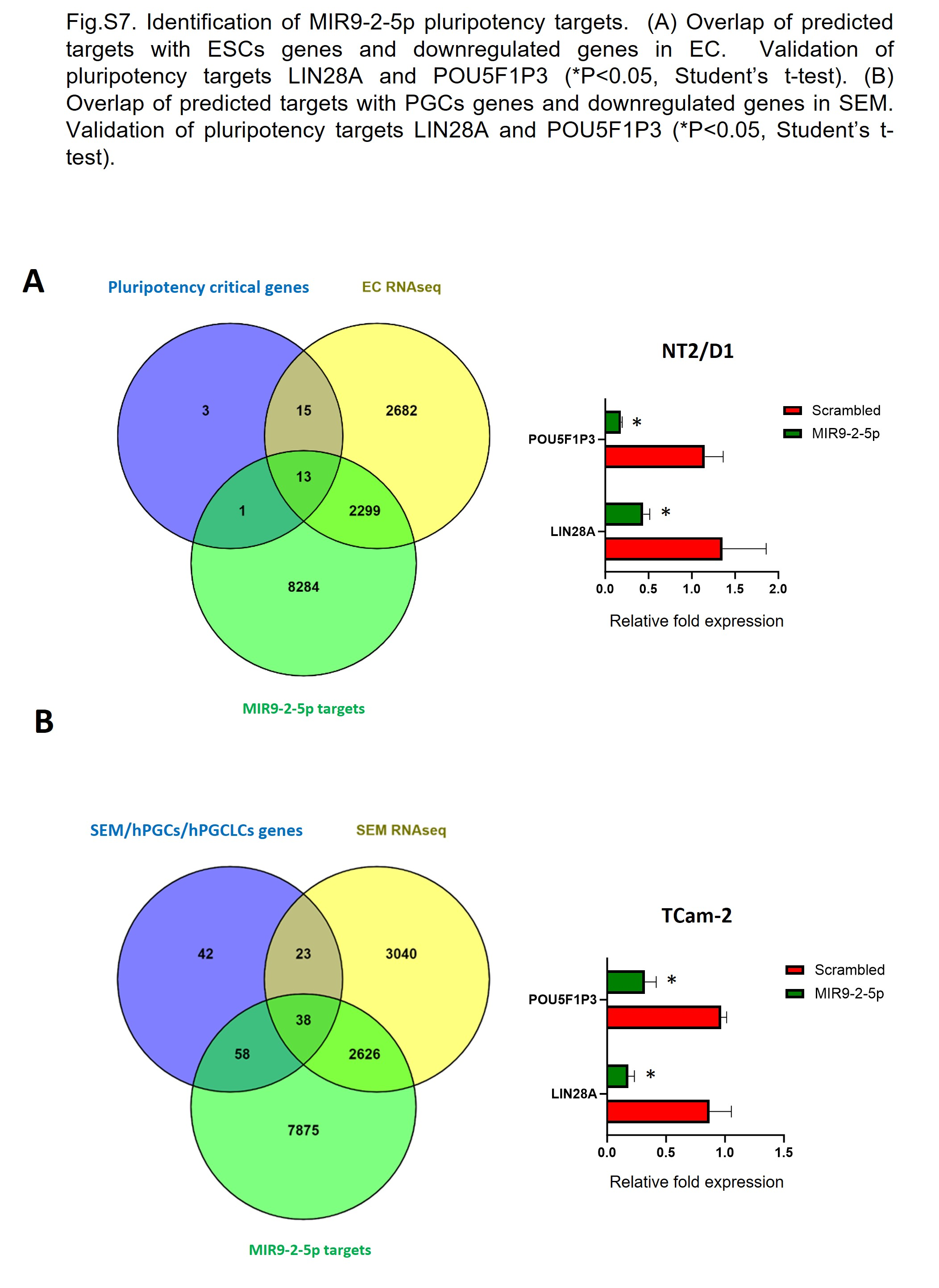

Supplement: Supplementary file 12 — Additional file 12: Fig. S7. Identification of MIR9-2-5p pluripotency targets. (A) Overlap of predicted targets with ESCs genes and downregulated genes in EC. Validation of pluripotency targets LIN28A and POU5F1P3 (*P < 0.05, Student’s t-test). (B) Overlap of predicted targets with PGCs genes and downregulated genes in SEM. Validation of pluripotency targets LIN28A and POU5F1P3 (*P < 0.05, Student’s t-test) [file 13287_2024_3724_MOESM12_ESM.png]
